# Examining the Impact of First Nations Status on the Relationship Between Diabetes and Cancer

**DOI:** 10.1089/heq.2019.0121

**Published:** 2020-05-18

**Authors:** Kathleen M. Decker, Pascal Lambert, Alain Demers, Erich V. Kliewer, Grace Musto, Natalie Biswanger, Brenda Elias, Donna Turner

**Affiliations:** ^1^ Department of Community Health Sciences, University of Manitoba, Winnipeg, Canada.; ^2^ Research Institute in Oncology and Hematology, CancerCare Manitoba, Winnipeg, Canada.; ^3^ Department of Epidemiology, CancerCare Manitoba, Winnipeg, Canada.; ^4^ Public Health Agency of Canada, Ottawa, Canada.; ^5^ Cancer Control Research, BC Cancer, Vancouver, Canada.

**Keywords:** cancer, diabetes, indigenous health

## Abstract

**Purpose:** This population-based study examined the relationship between diabetes and cancer and determined if this relationship was influenced by First Nations (FN) status.

**Methods:** In a matched case–cohort study, individuals 30–74 years of age diagnosed with diabetes during 1984–2008 in the province of Manitoba, Canada, with no cancer diagnosis before their diabetes diagnosis were matched to one diabetes-free control by age, sex, FN status, and residence. Flexible competing risk and Royston–Parmar regression models were used to compare cancer rates.

**Results:** Overall, 72,715 individuals diagnosed with diabetes were matched to controls. In all age groups, diabetes was related to an increased risk of cancer. The relationship between diabetes and any type of cancer was not influenced by FN status (i.e., there was no interaction between the diagnosis of diabetes and people's FN status for any age group). The only significant interaction between diabetes and FN status was for kidney cancer for individuals 60–74 years of age; diabetes increased the risk of kidney cancer for all other Manitobans (AOMs) but not for FN.

**Conclusions:** Diabetes increased the risk of cancer. The association was not modified by FN status except for kidney cancer where diabetes increased the risk for AOMs but not for FN.

## Introduction

Cancer rates among Canada's First Nations (FN), Inuit and Métis, peoples are increasing faster than cancer rates in the overall Canadian population.^1^ In Manitoba, the rate of breast cancer increased more rapidly among FN women than all other Manitoban (AOM) women from 1984 to 2008.^2^ The incidence of colorectal cancer among FN people also increased, and in 1999–2003, it surpassed the rate for AOM.^2^ In addition to the increasing cancer incidence rate, the burden of type 2 diabetes in FN is higher than in AOMs and growing.^3^ In Canada in 2008/09, the age-standardized prevalence of diabetes was 17.2% among FN individuals living on-reserve, 10.3% among FN individuals living off-reserve, 7.3% among Métis, and 5.0% in the nonindigenous population.^4^ In Manitoba in 2006, one in ten FN people had diabetes compared to one in sixteen AOMs.^5^ The age adjusted diabetes incidence was highest in FN females at 18 per 1000 in 2005/06, more than three times the incidence rate of 5 per 1000 in AOM females.^6^

There is evidence of an association between diabetes and specific types of cancer. Studies have found that individuals diagnosed with diabetes have an increased risk of colorectal, breast, and kidney cancer and a decreased risk of prostate cancer.^6–8^ The association between lung cancer and diabetes is less certain.^9,10^ The biological mechanism for the link between diabetes and cancer may be related to the indirect and direct effects of hyperinsulinemia, hyperglycemia, and chronic inflammation.^8^ However, the biological mechanisms for diabetes in FN may be different than for AOM,^11–15^ which may affect the association between diabetes and the risk of cancer.

### Problem to be addressed

Given the rapidly changing burden of cancer in FN, their higher risk of being diagnosed with diabetes, and the inequities in health experienced by Indigenous Peoples related to access to care, cultural safety, and a history of discrimination and trauma, a better understanding of the epidemiology and association between diabetes, cancer, and FN status is critical to plan effective prevention programs, policies, and service-based responses. This population-based study will address this problem by examining the relationship between diabetes and cancer and determining if this relationship is influenced by people's FN status.

## Materials and Methods

### Study design and population

A matched case–cohort study design was used.^16^ This study design and the models that were selected were used because they more precisely describe how risk changes over time. Individuals 30–74 years of age diagnosed with diabetes during 1984–2008 who did not have a cancer diagnosis before their diabetes diagnosis date (the index date) were matched to one diabetes-free control by age (±1 year), sex, FN status, and the first 2 characters of an individual's postal code. Urban included postal codes in the cities of Winnipeg and Brandon (i.e., postal codes that began with R1 or R9). All other postal codes were considered rural (i.e., postal codes that began with R0). Controls had to have no history of cancer at the index date. Individuals <30 years of age were excluded since the incidence of cancer is low in this age group.^17^

### Setting

The province of Manitoba, located in central Canada, has a population of ∼1.27 million; half the population lives in the capital city of Winnipeg. In 2016, 130,505 registered FN people were living in Manitoba representing 10.2% of the provincial population.^18^ “Registered” refers to FN individuals who, under the federal Indian Act, have treaty rights (also termed “status Indians”).^19^ In Manitoba, FN groups include Ojibway, Cree, Oji–Cree, Dakota, and Dene. The FN people in Manitoba reside in urban and rural areas, including 63 FN communities, some of which are isolated Northern communities.^18^ The FN population is younger than non-FN; the average age is 26.8 years for FN compared to 40.7 years for AOMs. Overall, 51.7% live on reserve and 48.3% live off reserve. A total of 30% of FN can conduct a conversation in an Indigenous language.^18^

### Data sources

Four data sources were used: the Federal Indian Registry, the Manitoba Health Population Registry (MHPR), the Manitoba Cancer Registry (MCR), and the Manitoba Diabetes Database (MDD). The Federal Indian Registry is the official record identifying Registered Indians in Canada. Permission was received from Indian and Northern Affairs Canada and the Assembly of Manitoba Chiefs to link the Indian Register to the MHPR. The MHPR includes all Manitoba residents covered by the Manitoba Health insurance program (99% of the population). Through a multistep data linkage process, registered FN individuals were identified in the MHPR creating a FN file.^20^ This deidentified FN file was then linked to the MCR using a scrambled personal health identification number unique to CancerCare Manitoba to identify individuals diagnosed with cancer. The MCR is a population-based registry of all cancers diagnosed in Manitoba residents since 1956. The quality of the MCR is excellent with consistently high levels of reporting completeness and histological verification.^21^

Individuals diagnosed with diabetes were identified using the MDD. The MDD was created by Manitoba Health using medical claims and hospital separation records that cited a diagnosis of diabetes (International Classification of Diseases, Ninth Revision, Clinical Modification code 250); it includes information on diabetes cases diagnosed since 1984. Individuals were defined as having diabetes based on the earliest date of two or more medical claims for diabetes within 2 years or one or more hospitalizations with a diagnosis of diabetes. The sensitivity of the database and validity of the diabetes case definition have been demonstrated.^22,23^ The database excludes individuals diagnosed with gestational diabetes, but does not make the distinction between types 1 and 2 diabetes. Individuals who move to or from the province that are identified as having diabetes were only included in the MDD for the years that they resided in the province of Manitoba.

### Statistical analyses

Descriptive statistics were used to examine the characteristics of the study individuals. The cumulative incidence of cancer by diabetes diagnosis and FN status, stratified by age group (30–44, 45–59, and 60–74 years) was described using cumulative incidence curves. The comp.risk function in the timereg R package (version 3.4.2), which is a flexible competing risk (CR) regression model using the subdistribution hazard ratio (SHR) approach, was used to compare cancer rates between diabetes status, FN status, and their interaction.^24^ A flexible model was used because the effect of diabetes on cancer incidence is expected to vary with time due to the increase in cancer case finding (due to increased diagnostic testing and case ascertainment) immediately following a diabetes diagnosis.^25^ The flexible model eliminates the need to exclude cancers diagnosed during a randomly defined time period after a diabetes diagnosis (the method used in many previous studies).^26^ Plots of the time-varying SHRs postdiagnosis and 95% confidence intervals (CIs) were produced to demonstrate the effect of diabetes. The analysis was adjusted for age at diagnosis, sex, and area of residence. Models were run for all cancers combined as well as separately for colorectal, lung, breast, prostate, and kidney cancers. For the CR models, proportional hazard assumption was tested for variables (other than diabetes) using observed and simulated test processes. Linearity was tested using the quartile method.^27^

A Royston–Parmar (RP) regression model was also used to compare cancer rates between groups for the time individuals were alive (i.e., death was censored).^28^ This model was used because FN individuals are expected to have higher rates of death particularly at younger ages, which eliminates the possibility of being diagnosed with cancer at an older age. The RP regression model is a flexible parametric survival model that uses cubic splines to account for the effect of time on a continuous scale. An interaction between a factor and time allows the calculation of time-varying estimates for that factor. Plots of time-varying hazard ratios (HRs), including 95% CIs were used to display the time-varying effects of diabetes. We censored for death (the CR) and ran RP models for all cancers combined as well as separately for colorectal, lung, breast, prostate, and kidney cancers. We used four knots for time and three knots for the interaction between time and diabetes. The number of knots that represented the best fit was determined using Akaike information criterion scores. Knots are used to calculate the values in the splines, which are then entered into the regression model to account for the effect of diabetes which varies over time. Plots were produced to illustrate the time-varying effect of diabetes, which is possible when including the main effects of diabetes, time, and the interaction between the two. The analysis was adjusted for age at diagnosis, sex, and area of residence. For RP models, the proportional hazard assumption was tested for all variables (other than diabetes) using time interaction terms. Linearity was tested using restricted cubic splines. The stpm2 STATA (version 14.2) procedure was used.^29^ Both CR analyses and RP analyses included models with and without an interaction between diabetes and FN status.

Ethical approvals were received from the University of Manitoba Health Research Ethics Board, the Manitoba Health's Health Information Privacy Committee, CancerCare Manitoba's Research Impact Committee, and the Assembly of Manitoba Chief's Health Information and Research Governance Committee.

## Results

Overall, 72,715 individuals diagnosed with diabetes 30–74 years of age matched to controls were included (Table 1). For individuals 30–44 and 45–59 years of age, a greater percentage of FN diabetes cases were females while a greater percentage of AOM diabetes cases were males. For the 60–74 years of age, males represented a greater percentage of the diabetes cases for FN and AOM. The median follow-up time was 6.9 years. A total of 896 FN individuals and 9771 AOM were diagnosed with cancer (Table 2).

**Table 1.** Characteristics of Study Individuals by Age Group, Diabetes Diagnosis, and First Nations Status, Manitoba, 1984–2008 (*n*=145,430)

	30–44 (n=31,820)				45–59 (n=61,216)				60–74 (n=52,394)			
Age (years)	Diabetes (n=15,910)		No diabetes (n=15,910)		Diabetes (n=30,608)		No diabetes (n=30,608)		Diabetes (n=26,197)		No diabetes (n=26,197)	
	FN	AOM	FN	AOM	FN	AOM	FN	AOM	FN	AOM	FN	AOM
Age (mean, SD)	37.4 (4.24)	38.7 (4.09)	37.4 (4.27)	38.7 (4.16)	51.2 (4.19)	52.4 (4.22)	51.1 (4.26)	52.4 (4.30)	65.3 (4.10)	66.5 (4.27)	65.3 (4.17)	66.5 (4.35)
Sex												
Female	2498 (54.7)	5268 (46.4)	2498 (54.7)	5268 (46.4)	2008 (53.0)	11223 (41.9)	2008 (53.0)	11223 (41.9)	658 (47.0)	11437 (46.1)	658 (47.0)	11437 (46.1)
Male	2068 (45.3)	6076 (53.6)	2068 (45.3)	6076 (53.6)	1783 (47.0)	15594 (58.1)	1783 (47.0)	15594 (58.1)	742 (53.0)	13360 (53.9)	742 (53.0)	13360 (53.9)
Residence^a^												
Brandon	118 (2.6)	473 (4.2)	118 (2.6)	473 (4.2)	65 (1.71)	1174 (4.4)	65 (1.71)	1174 (4.4)	22 (1.6)	1152 (4.6)	22 (1.6)	1152 (4.6)
Other rural	3312 (72.5)	3443 (30.4)	3312 (72.5)	3443 (30.4)	2817 (74.3)	8978 (33.5)	2817 (74.3)	8978 (33.5)	1133 (80.9)	9437 (38.1)	1133 (80.9)	9437 (38.1)
Winnipeg	1136 (24.9)	7428 (65.5)	1136 (24.9)	7428 (65.5)	909 (24.0)	16665 (62.1)	909 (24.0)	16665 (62.1)	245 (17.5)	14208 (57.3)	245 (17.5)	14208 (57.3)

^a^ Categorized using the 6-digit postal code.

AOM, all other Manitobans; FN, First Nations; SD, standard deviation.

**Table 2.** Characteristics of Individuals Diagnosed with Cancer by Age Group, Diabetes Diagnosis, and First Nations Status, Manitoba, 1984–2008 (*n*=10,667)

Age (years)	Diabetes		No diabetes	
	FN	AOM	FN	AOM
30–44	96	269	68	220
45–59	230	1867	163	1474
60–74	171	3484	168	2457

**FIG. 1.**
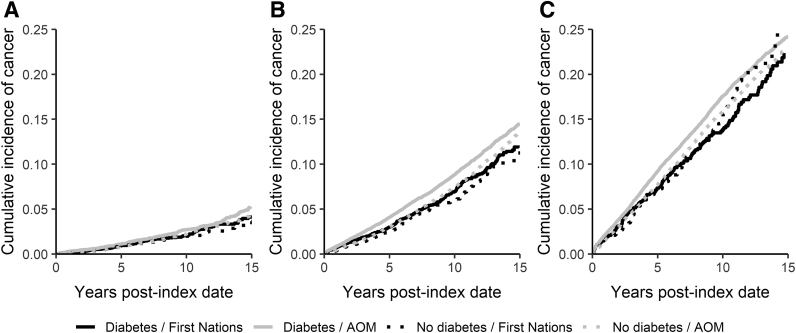
Cumulative incidence of all cancers by diabetes diagnosis for FN and AOM, **(A)** 30–44 years of age, **(B)** 45–59 years of age, **(C)** 60–74 years of age. AOM, all other Manitoban; FN, First Nations.

For age groups 30–44 and 45–59 years, the cumulative cancer incidence was highest for AOM with diabetes and lowest for FN without diabetes (Fig. 1A, B). For individuals 60–74 years of age, the cumulative cancer incidence for FN without diabetes surpassed AOM with diabetes after ∼10 years of follow-up (Fig. 1C).

The risk of cancer by age group, diabetes diagnosis, and FN status with death as a CR was examined (Supplementary Table S1). Diabetes was included in a flexible CR model to produce a time-varying coefficient, the results of which were plotted. The elevated risk observed immediately after the index date in all age groups is related to the additional workup that occurs after a diabetes diagnosis. For individuals 30–44 years of age, there was a short-term elevated risk of cancer immediately following a diabetes diagnosis, but overall, the effect of diabetes on cancer risk was not significant (Supplementary Fig. S1A). For individuals 45–59 years of age, there was a significant effect of diabetes on cancer risk, which remained significantly elevated after ∼10 to 15 years after diagnosis (Supplementary Fig. S1B). For the oldest age group (60–74 years of age), there was a significant effect of diabetes on cancer incidence up to 10–15 years after diagnosis (Supplementary Fig. S1C). For all age groups, the relationship between diabetes and cancer was not influenced by FN status (i.e., there was no interaction between the diagnosis of diabetes and people's FN status for any age group).

CR models were also run for colorectal, lung, breast, prostate, and kidney cancers for individuals 45–59 and 60–74 years of age. Individuals 30–44 years of age were excluded due to the small number of cancer cases. The only significant interaction between diabetes and FN was for kidney cancer for individuals 60–74 years of age (SHR 0.37, 95% CI 0.15–0.94, *p*<0.036); the relationship between diabetes and kidney cancer was weaker among FN individuals compared to AOMs.

**FIG. 2.**
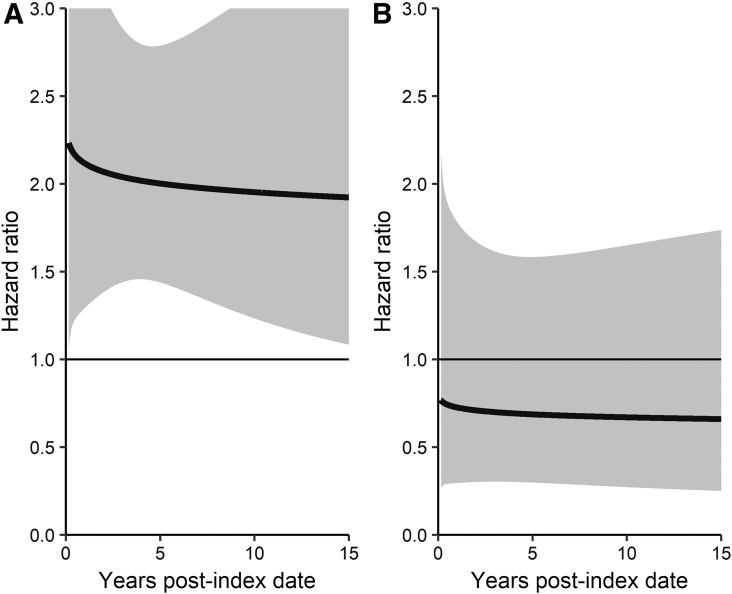
Royston–Parmar regression time-varying plot of diabetes and kidney cancer and interaction between diabetes, 60–74 years of age, **(A)** AOM, **(B)** FN.

The risk of being diagnosed with any type of cancers for individuals for the time they were alive (the RP model where death was censored) was similar to that found in the CR analysis (Supplementary Table S2). In all age groups, the effect of diabetes was related to an increased risk of cancer incidence (Supplementary Fig. S2A–C). However, there was no significant interaction between diabetes and FN status for all cancers combined. The risk for colorectal, lung, breast, and prostate cancers was similar as that found for all cancers using the RP model. For kidney cancer in individuals 60–74 years of age, the effect of diabetes on kidney cancer risk did depend on FN status (HR 0.34, 95% CI 0.14–0.83, *p*=0.017). Similarly to the CR model, the plots show that for AOMs, diabetes increased the risk of kidney cancer for AOMs (Fig. 2A) but not for FN (Fig. 2B).

## Discussion

We found that although the overall cancer risk was not different for FN or AOM individuals, there was a significant interaction between diabetes and FN on the risk of kidney cancer. For FN individuals, a diagnosis of diabetes did not increase the risk of kidney cancer but for AOM, a diagnosis of diabetes did increase the risk of kidney cancer.

However, many risk factors for kidney cancer (tobacco exposure, obesity, and hypertension) are prevalent among FN in Canada and the incidence of diabetes is higher in this population.^30–34^ Previous research has also found a significantly increased risk of kidney cancer in individuals diagnosed with diabetes.^35,36^ Therefore, it is possible that the higher prevalence of these risk factors for kidney cancer in the FN population reduced the heterogeneity between individuals with and without diabetes in this study leading to the nonsignificant difference between diabetes and no diabetes for FN.

This study also found that people with diabetes are at increased risk of being diagnosed with cancer, but the risk varied over time. Regardless of age, the risk of cancer was highest within the first few years following a diabetes diagnosis, which is most likely due to ascertainment bias. Nevertheless, the risk of cancer remained elevated for individuals 45–74 years of age for several years. Our results add to the growing body of epidemiological evidence supporting the link between diabetes and the incidence of some types of cancer.^37–39^ The present study emphasizes the importance of including a time-varying component in analyses to account for the impact of increased diagnostic testing on cancer incidence following a diabetes diagnosis.^26,37^

The strengths of this study include the linkage of the Federal Indian Registry to the MHPR to accurately identify FN individuals, the use of a population-based cancer registry, and the use of time-varying models instead a randomly selected washout time period. Several limitations should be considered in the interpretation of our findings. Due to the high number of analyses that were run, the significant interaction between diabetes, FN status, and kidney cancer could be due to a random association. The MDD depends on diabetes cases being recognized, diagnosed, and recorded through interactions with the health care system. Using administrative data likely underestimates the incidence and prevalence of diabetes.^23,40^ We were not able to differentiate between types 1 and 2 diabetes. However, ∼90% to 95% diabetes cases are type 2 diabetes and type 1 diabetes is very uncommon among FN people.^41^ We were not able to include environmental or lifestyle risk factors for cancer or distinguish between FN who live on versus off reserve, which could have an impact on the rate of cancer diagnosis. Finally, differences in the detection of cancer between FN and AOMs may be present due to differential access to screening or diagnostic tests. For example, if FN individuals have, on average, fewer diagnostic tests performed, they may have fewer cancers diagnosed. This detection bias may have resulted in either overestimating or underestimating the relationship between diabetes, FN status, and cancer.

## Conclusions

There is no evidence that diabetes is associated with a strongly increased risk of any cancer type for FN when compared to AOM except for kidney cancer where diabetes increased the risk for AOMs but not for FN. Regardless, FN persons experience a higher risk of diabetes, the incidence of cancer is increasing, and cancer mortality is often higher. These issues must be addressed in culturally appropriate ways that improve access to care and reduce health inequities.

## Supplementary Material

Supplemental data

Supplemental data

Supplemental data

Supplemental data

## Abbreviations Used

AOM: all other Manitoban
CI: confidence interval
CR: competing risk
FN: First Nations
HR: hazard ratio
MCR: Manitoba Cancer Registry
MDD: Manitoba Diabetes Database
MHPR: Manitoba Health Population Registry
RP: Royston–Parmar
SD: standard deviation
SHR: subdistribution hazard ratio
