# Supplementary material for: Examining the Impact of First Nations Status on the Relationship Between Diabetes and Cancer
Source: Health Equity. 2020 May 18;4(1):211–7. doi: 10.1089/heq.2019.0121 (PMC7241056; doi:10.1089/heq.2019.0121)
Supplement: Supplemental data [file Supp_TableS1.pdf]

Supplementary Table S1. Risk of Being Diagnosed with Any Cancer by Diabetes Diagnosis, First Nations Status, and Age Group

			Main effects		Main effects with interaction†	
Age (years)	Variable	Comparison	SHR (95% CI)	p	SHR (95% CI)	p
30–44	Diabetes	Yes/no	Not shown	0.045	Supplementary Figure S1A	0.137
	FN status	FN/AOM	0.99 (0.79–1.24)	0.926	0.84 (0.61–1.16)	0.286
	Diabetes × FN status				1.27 (0.84–1.90)	0.256
45–59	Diabetes	Yes/no	Not shown	< 0.001	Supplementary Figure S1B	< 0.001
	FN status	FN/AOM	0.89 (0.78–1.00)	0.059	0.92 (0.75–1.13)	0.428
	Diabetes × FN status				0.94 (0.74–1.21)	0.651
60–74	Diabetes	Yes/no	Not shown	< 0.001	Supplementary Figure S1C	< 0.001
	FN status	FN/AOM	0.92 (0.81–1.05)	0.208	0.94 (0.81–1.22)	0.956
	Diabetes × FN status				0.86 (0.66–1.13)	0.282

Notes: Plots for the main effects only are not shown as they are the same as the plots that include the interaction.
*Time-varying ratios.
†Adjusted for age, sex, and area of residence.
AOM, all other Manitobans; CI, confidence interval; FN, First Nations; SHR, subdistribution hazard ratio.
