# Supplementary material for: Examining the Impact of First Nations Status on the Relationship Between Diabetes and Cancer
Source: Health Equity. 2020 May 18;4(1):211–7. doi: 10.1089/heq.2019.0121 (PMC7241056; doi:10.1089/heq.2019.0121)
Supplement: Supplemental data [file Supp_FigS1.pdf]

Supplementary Data

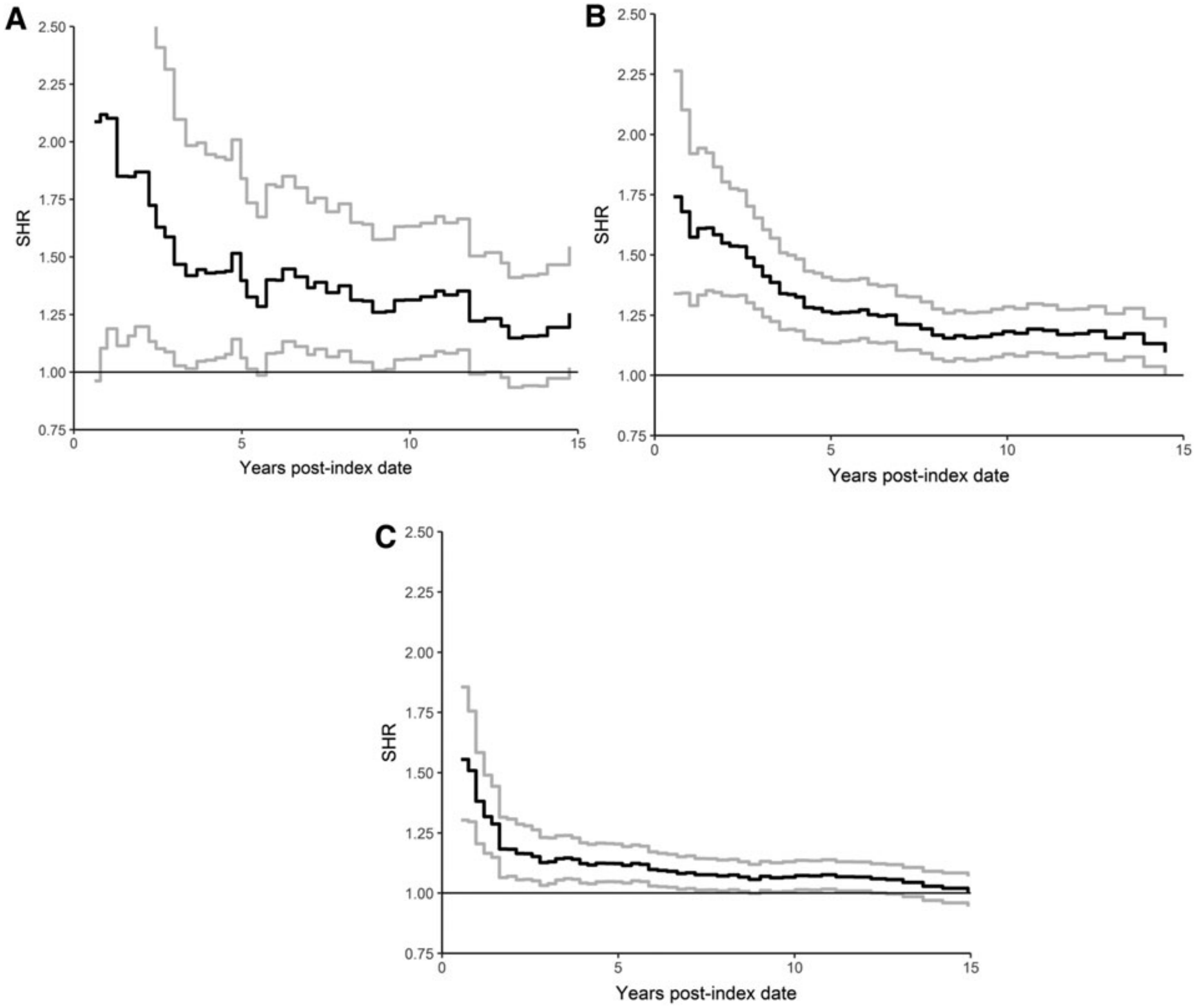

SUPPLEMENTARY FIG. S1. Competing risk regression time-varying plot for DM and all cancers, main effects, and interactions, **(A)** 30–44 years of age, **(B)** 45–59 years of age, **(C)** 60–74 years of age. DM, diabetes mellitus; SHR, subdistribution hazard ratio.
