# Supplementary material for: Examining the Impact of First Nations Status on the Relationship Between Diabetes and Cancer
Source: Health Equity. 2020 May 18;4(1):211–7. doi: 10.1089/heq.2019.0121 (PMC7241056; doi:10.1089/heq.2019.0121)
Supplement: Supplemental data [file Supp_TableS2.pdf]

**Supplementary Table S2. Risk of Being Diagnosed with Any Cancer by Diabetes Mellitus Status, First Nations Status, Age Group, and Time with Death Censored**

| Age (years) | Variable | Comparison | Main effects HR (95% CI) | Main effects *p* | Main effects with interaction* HR (95% CI) | Main effects with interaction* *p* |
|---|---|---|---|---|---|---|
| 30–44 | Diabetes | Yes/no | 1.37 (1.13–1.66) | 0.001 | 1.38 (1.12–1.71) | 0.003 |
| 45–59 | FN status | FN/AOM | 0.92 (0.76–1.10) | 0.354 | 0.93 (0.71–1.22) | 0.602) |
| | Diabetes × FN status | | | | 0.97 (0.69–1.38) | 0.882 |
| | Time | ′† | 3.72 (3.19–4.35) | < 0.001 | 3.72 (3.19–4.35) | < 0.001 |
| | | ″ | 0.84 (0.75–0.94) | | 0.84 (0.75–0.94) | |
| | | ‴ | 0.92 (0.89–0.95) | | 0.92 (0.89–0.95) | |
| | Diabetes × time | ′ | 0.87 (0.72–1.06) | 0.371 | 0.87 (0.72–1.06) | 0.371 |
| | | ″ | 0.97 (0.85–1.11) | | 0.97 (0.85–1.11) | |
| | Diabetes | Yes/no | 1.33 (1.23–1.44) | < 0.001 | 1.33 (1.22–1.44) | < 0.001 |
| | FN status | FN/AOM | 0.88 (0.78–0.98) | 0.018 | 0.87 (0.74–1.04) | 0.119 |
| | Diabetes × FN status | | | | 1.00 (0.81–1.25) | 0.979 |
| | Time | ′ | 3.54 (3.32–3.77) | < 0.001 | 3.54 (3.32–3.77) | < 0.001 |
| | | ″″ | 0.89 (0.84–0.93) | | 0.89 (0.84–0.93) | |
| | | ‴ | 0.96 (0.94–0.97) | | 0.96 (0.94–0.97) | |
| | Diabetes × time | ′ | 0.85 (0.78–0.92) | < 0.001 | 0.85 (0.78–0.92) | < 0.001 |
| | | ″ | 0.96 (0.91–1.01) | | 0.96 (0.91–1.01) | |
| 60–74 | Diabetes | Yes/no | 1.21 (1.15–1.28) | < 0.001 | 1.22 (1.15–1.29) | < 0.001 |
| | FN status | FN/AOM | 0.99 (0.88–1.11) | 0.884 | 1.03 (0.87–1.22) | 0.711 |
| | Diabetes × FN status | | | | 0.93 (0.74–1.17) | 0.525 |
| | Time | ′ | 3.45 (3.29–3.61) | < 0.001 | 3.45 (3.29–3.61) | < 0.001 |
| | | ″ | 0.97 (0.93–1.01) | | 0.97 (0.93–1.01) | |
| | | ‴ | 0.98 (0.97–0.99) | | 0.98 (0.97–0.99) | |
| | Diabetes × time | ′ | 0.85 (0.80–0.90) | < 0.001 | 0.85 (0.80–0.90) | < 0.001 |
| | | ″ | 0.91 (0.87–0.95) | | 0.91 (0.87–0.95) | |

*Adjusted for age, sex, and area of residence

†Splines.

HR, hazard ratio.
