# Supplementary material for: Examining the Impact of First Nations Status on the Relationship Between Diabetes and Cancer
Source: Health Equity. 2020 May 18;4(1):211–7. doi: 10.1089/heq.2019.0121 (PMC7241056; doi:10.1089/heq.2019.0121)
Supplement: Supplemental data [file Supp_FigS2.pdf]

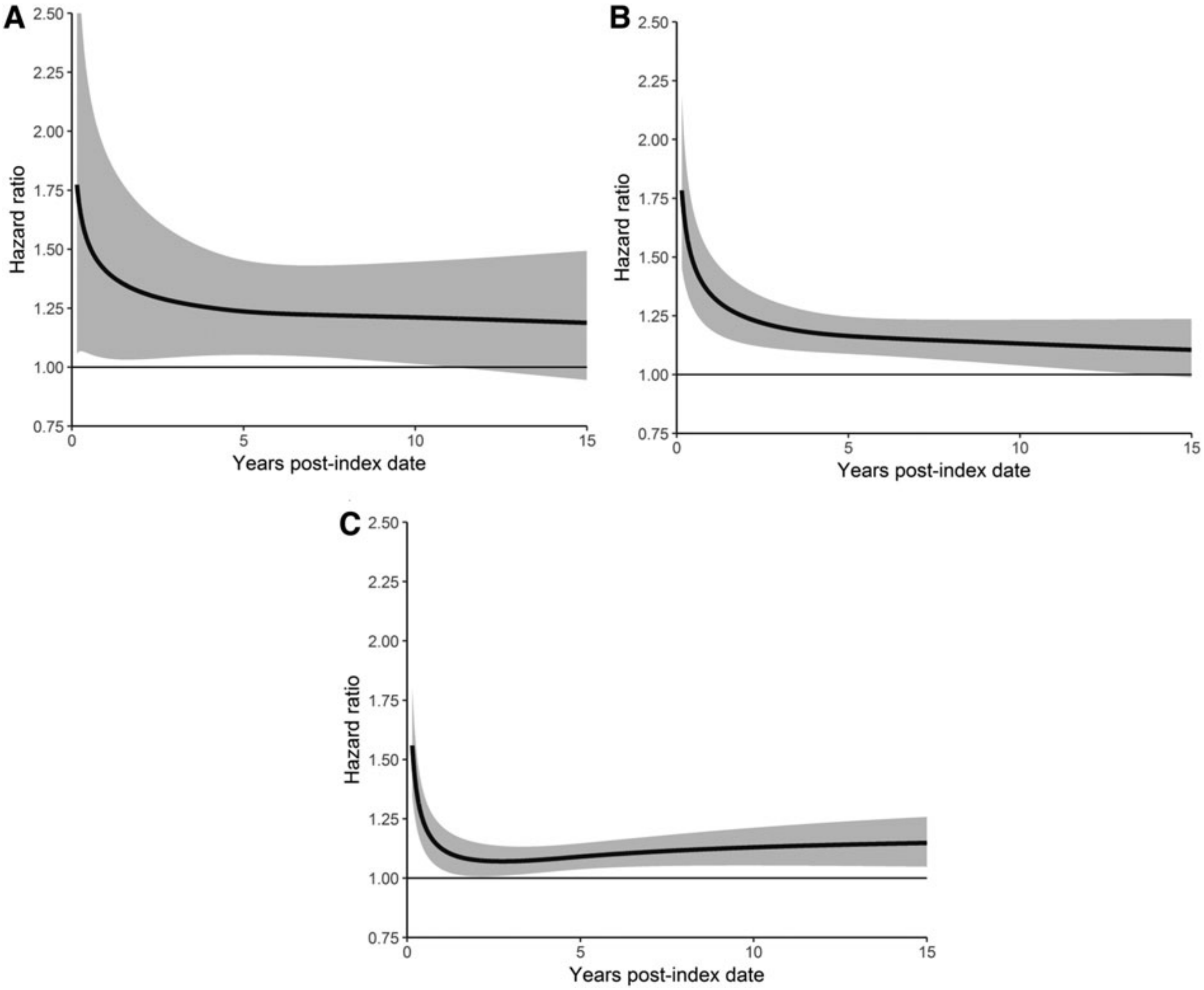

SUPPLEMENTARY FIG. S2. Royston–Parmar regression time-varying plot of DM and all cancers, main effects, and interactions, **(A)** 30–44 years of age, **(B)** 45–59 years of age, **(C)** 60–74 years of age.
